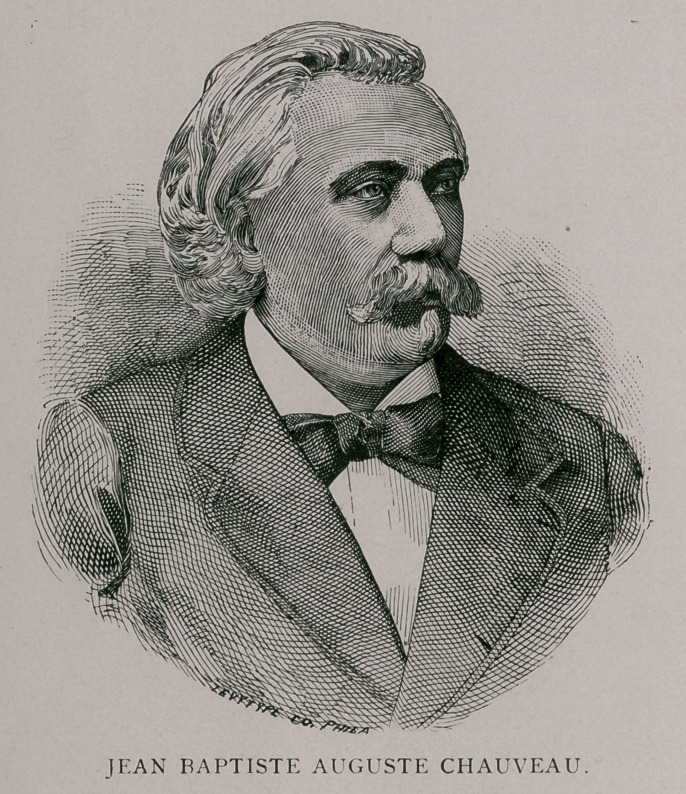# Jean Baptiste Auguste Chauveau

**Published:** 1887-04

**Authors:** 


JEAN BAPTISTE AUGUSTE CHAUVEAU.

### Art. XIX.—JEAN BAPTISTE AUGUSTE CHAUVEAU.

Jean Baptiste Auguste Chauveau was born at Villeneuve-la-Guyard (Yonne), France, on the 21st of November, 1827. Locality and climate, which in the Midi keep the olive trees green the year round, and furnish orange flowers in mid-winter, left their trace on M. Chauveau, and bred in him the genial, warm-hearted, hospitable nature belonging to men of the South. Educated at the Royal Veterinary School at Alfort (Seine), M. Chauveau received with fervor the accurate scientific teachings of the colder blooded Frenchmen of the North, and laid the foundation for that minute application to details, and ready appreciation and comprehension of complex subjects, which have made him a leader of the veterinary profession, and a peer of all scientists. Immediately upon receiving his diploma as veterinarian at Alfort, M. Chauveau was appointed Chief of the Anatomical Service at the Veterinary School at Lyons, France, (1848).

The commencement of his career was marked by publications of importance on comparative anatomy, and on the physiology of the heart and nerves. Upon a vacancy being created by the promotion of M. Lecoq, M. Chauveau was nominated to a Professorship in 1863. He became Director of the Lyons Veterinary School in 1876, and was made Professor of Experimental Medicine in the Faculty of Medicine at Lyons, in 1877. Upon the death of the great *M. Bouley*, M. Chauveau was appointed Inspector General of the Veterinary Schools of France, in 1886.

M. Chauveau is probably best known to the English speaking members of the Veterinary profession as the author of the standard text-book, "Anatomie Comparee des Animaux Domestiques," first edition, 1854; translated into English by Mr. George Fleming. The second and third editions of the Anatomy, published in 1872–1877, with the collaboration of M. Arloing, by D. Appleton & Co., New York, were but examples of M. Chauveau's wonderful facility for teaching and developing in those who had the pleasure of working under him, the love of truthful observation and ability of expression, which are inherent parts of himself; there is probably no living scientist whose work is so minute, and whose reports can be accepted as so absolutely accurate and correct. In his laboratory at Lyons a visitor is made to feel like an ambassador, a student like a son, and the veterinarian who has been thrown personally with M. Chauveau, recognizes that his profession is the greatest in opportunity and future of any art which man can follow.

M. Chauveau is correspondent of the Academie des Sciences, Laureat de l'Institut, four prizes.

*Associate member*—Academy of Medicine (Paris); Society of Agriculture; Society of Biology; Central Society of Veterinary Medicine; Society of Vet. Med. of St. Petersburg.

*Honorary member*—Academy of Medicine (Belgium); Academy of Medicine, (Turin); University of Edinburg (LL. D.); Society of Medicine (London); Society of Medicine (St. Petersburg), etc., etc.

He is author of works on Rinderpest, 1865. Vaccinia and

Variola, 1866–1884. Septicæmia, 1872. Virus and Prophylactic Inoculations, 1868–1881–82. Attenuation of Viruses, 1880–81–82–83–84. Tuberculosis, nine papers, 1884. Anthrax, seven papers, 1879–80. Pyæmia and Puerpural Septicæmia, Hospital Gangrene, 1884. Anatomy—Comparative, genital organs of the cow, growth of horn. Mechanism of the heart and circulation, nineteen papers, 1855 to 1870. Hepatic Glycogeny, four papers, 1856–57. Function Spinal Marrow and General Physiology of Nerves, seventeen papers, 1857–1878. Semiology—Respiration, Circulation, six papers, 1858–60.

## Figures and Tables

**Figure f1:**